# Aortic Stiffness as a Surrogate Endpoint to Micro- and Macrovascular Complications in Patients with Type 2 Diabetes

**DOI:** 10.3390/ijms17122044

**Published:** 2016-12-06

**Authors:** Claudia R. L. Cardoso, Gil F. Salles

**Affiliations:** Department of Internal Medicine, School of Medicine and University Hospital Clementino Fraga Filho, Universidade Federal do Rio de Janeiro, Rua Rodolpho Rocco 255, Cidade Universitária, Rio de Janeiro-RJ 21941-901, Brazil; claudiacardoso@hucff.ufrj.br

**Keywords:** arterial stiffness, type 2 diabetes, carotid-femoral pulse wave velocity, microvascular and macrovascular complications

## Abstract

Increased aortic stiffness has been recognized as a predictor of adverse cardiovascular outcomes in some clinical conditions, such as in patients with arterial hypertension and end-stage renal disease, in population-based samples and, more recently, in type 2 diabetic patients. Patients with type 2 diabetes have higher aortic stiffness than non-diabetic individuals, and increased aortic stiffness has been correlated to the presence of micro- and macrovascular chronic diabetic complications. We aimed to review the current knowledge on the relationships between aortic stiffness and diabetic complications, their possible underlying physiopathological mechanisms, and their potential applications to clinical type 2 diabetes management.

## 1. Introduction

The role of aortic stiffness in the pathogenesis of cardiovascular diseases has been increasingly recognized in the last decade [1,2]. Arterial stiffness relies on structural and geometric properties of the arterial wall and on the distending pressure; aging and blood pressure are its main related factors [3]. Pulse wave velocity (PWV) is the most largely employed technique to evaluate arterial stiffness. Although PWV can be measured on any artery or between any artery sites, carotid–femoral PWV, representing stiffness of the aorta and iliofemoral axes, is the most widely used index of arterial stiffness [4,5]. Carotid–femoral PWV has been shown to be a predictor of cardiovascular outcomes, over and beyond traditional risk factors in several longitudinal follow-up studies, in different populations [6,7,8,9,10]. Otherwise, the predictive values of PWV measurements on other arterial segments are not yet so extensively evaluated. Currently, there is epidemiological evidence that the brachial–ankle PWV, a derivative technique that measures PWV between brachial and ankle arteries, may be a predictor of cardiovascular events in East Asian populations [11]. The clinical utility of the cardio-ankle vascular index (CAVI) measurement, another potential measure of arterial stiffness, is presently under investigation [12]. Carotid–femoral PWV is currently regarded as the gold standard mode of measuring central (aortic) stiffness [4,13].

Type 2 diabetic patients have increased aortic stiffness [14,15,16] and are at specially increased risk for cardiovascular morbidity and mortality. This high cardiovascular risk is not entirely due to grouping of conventional risk factors, and increased aortic stiffness may be one of the mechanisms connecting diabetes to increased cardiovascular morbidity and mortality [17]. In addition, the prognostic importance of aortic stiffness for cardiovascular endpoints has been shown in different clinical conditions [6,7], including type 2 diabetes [8,9], and in a novel meta-analysis [10] independent of established cardiovascular risk factors. Although relationships between increased aortic stiffness and the presence of microvascular complications have been largely shown in cross-sectional analyses [18,19,20,21,22,23,24,25,26,27,28,29,30,31,32], the importance of increased aortic stiffness for the onset and progression of microvascular complications is much less explored. Up to now, two prospective follow-up cohorts of type 2 diabetic patients investigated the prognostic importance of aortic stiffness, both by carotid–femoral PWV measurement, for microvascular outcomes [33,34]. The first study showed that baseline aortic stiffness was associated with incident albuminuria and with the rate of decline in glomerular filtration rate in type 2 diabetic patients [33]. The second one demonstrated that baseline aortic stiffness predicts future installation and progression of peripheral diabetic neuropathy [34].

Therefore, we review the recent evidence regarding aortic stiffness as a predictor of cardiovascular morbidity and mortality and of development and progression of microvascular complications in patients with type 2 diabetes. Furthermore, we address possible physiopathological mechanisms regarding the associations between increased aortic stiffness and the occurrence of macro- and microvascular complications in type 2 diabetes.

## 2. Increased Aortic Stiffness as a Predictor of Future Cardiovascular Complications Occurrence in Type 2 Diabetes

Increased aortic stiffness, measured by carotid–femoral PWV, has been well-demonstrated to represent an independent predictor of future major cardiovascular events occurrence in several clinical settings, such as in arterial hypertensive patients [6,35], in patients with end-stage renal disease [36], in elderly individuals [7,37], and in population-based samples [38,39]. Also, two recent meta-analyses confirmed these observations [10,40]. Nevertheless, there are only three studies that evaluated the prognostic value of increased aortic stiffness in patients with type 2 diabetes [8,9,41], which we have previously reviewed [42]. Two of them included high risk type 2 diabetic patients originating from tertiary care centers [8,41] and the third was performed in primary care clinics [9]. The first pioneering study [41] used a non-validated simultaneous aortic arc–abdominal aorta Doppler flow probe to evaluate aortic PWV. It evaluated 397 type 2 diabetic patients and observed 179 all-cause deaths over a median follow-up of 10 years. It demonstrated that a 1 m/s increase in aortic PWV was associated with an 8% excess risk (95% confidence interval: 3% to 14%) of all-cause mortality, after statistical adjustments for age, gender and systolic blood pressure [41]. The second study, the Rio de Janeiro type 2 diabetes (RIO-T2D) cohort study, was also performed in high-risk type 2 diabetic patients [8]. We evaluated 565 type 2 diabetic patients and observed 88 major cardiovascular events and 76 all-cause deaths over a median follow-up of 6 years. We showed that an increment of 1 m/s in carotid–femoral PWV was a predictor of future cardiovascular events, with a hazard ratio of 1.13 (95% confidence interval: 1.03–1.23), over and beyond other conventional cardiovascular risk markers, the presence of micro- and macrovascular complications, metabolic control and ambulatory blood pressures [8]. Also, increased aortic stiffness, defined as a carotid–femoral PWV > 10 m/s, was associated with nearly a two-fold excess cardiovascular risk (hazard ratio: 1.92; 95% confidence interval: 1.16–3.18). This study further demonstrated that the inclusion of aortic stiffness improved cardiovascular risk stratification in relation to the model with traditional risk factors. The predictive power of aortic stiffness for total cardiovascular outcomes was more marked in younger subjects, in patients with microvascular complications and with inadequate glycemic control. However, in our study [8], increased aortic stiffness did not predict all-cause mortality (hazard ratio: 1.06; 95% confidence interval: 0.95–1.17; estimated for increments of 1 m/s in carotid–femoral PWV). Based on these results, we suggested that aortic stiffness assessment should be routinely performed in cardiovascular risk stratification of high-risk type 2 diabetic patients [8]. The more recent study [9] evaluated 627 lower-risk type 2 diabetic patients in primary care, and observed 45 major cardiovascular events over a median follow-up of 8 years. It confirmed the aortic stiffness prognostic role for the prediction of cardiovascular events also in lower risk type 2 diabetic patients, with an estimated hazard ratio of 1.14 (95% confidence interval: 1.003–1.30) for each 1 m/s increase in carotid–femoral PWV, after statistical adjustment for classic cardiovascular risk factors and glycemic control. Most importantly, it showed that the prognostic value of increased aortic stiffness was still evident in those patients with normal blood pressure and adequate glycemic control [9]. None of these previous studies [8,9,41] showed any interaction between gender and the prognostic value of increased aortic stiffness, suggesting that aortic stiffness equally predicts worse cardiovascular prognosis in both men and women with type 2 diabetes. This observation has also been demonstrated in previous meta-analyses [10,40].

These studies enforce the importance of aortic stiffness as a predictor of cardiovascular morbidity and mortality in patients with type 2 diabetes. Moreover, carotid–femoral PWV measurement is a relatively simple, moderate-cost method, with a well-standardized procedure and equipment [4,43] and with a validated and accepted range of normal values [44]. So, based on current knowledge, we strongly recommend that aortic stiffness assessment should be included in the clinical management of type 2 diabetes [42].

## 3. Relationships between Increased Aortic Stiffness and Microvascular Complications Occurrence

The presence of diabetic microvascular complications (retinopathy, nephropathy and neuropathy) have all been widely reported as independently associated with higher arterial stiffness in cross-sectional analyses [18,19,20,21,22,23,24,25,26,27,28,29,30,31,32]. Table 1 summarizes the main studies that evaluated associations between arterial stiffness indices and diabetic microvascular complications. The presence of diabetic peripheral neuropathy has been reported to be associated with increased arterial stiffness measured by different methods, such as carotid–femoral PWV [20], ankle–brachial PWV [22,23] and CAVI [21,30] in cross-sectional studies with type 2 diabetic patients. Otherwise, associations between cardiovascular dysautonomia and increased aortic stiffness are less explored. A study including 45 type 2 diabetic patients [24], and a larger one with 676 patients with type 1 diabetes [25] demonstrated associations between increased aortic stiffness and cardiovascular dysautonomia, both using carotid–femoral PWV measurement as the method of aortic stiffness assessment. Diabetic retinopathy has also been associated with increased arterial stiffness evaluated by different methods of measurement, including PWV [20,26] and augmentation index [19], an indirect parameter of central aortic stiffness. Relationships between abnormal albuminuria and increased arterial stiffness have also been demonstrated, using different PWV measurements: carotid–femoral [18,20,27,28], ankle–brachial [29] and CAVI [30]. Some of the studies reported that the relationship between increased aortic stiffness and the presence of microalbuminuria persisted after statistical adjustment for blood pressure levels [18,28], but others did not [27], suggesting that at least part of this association might be mediated by increased blood pressure levels. Although renal failure was independently associated with increased aortic stiffness in a study including subjects with and without type 2 diabetes, when analyzing separately the subgroup of diabetic patients, only age and mean arterial blood pressure were independently related to increased arterial stiffness evaluated by carotid–femoral PWV [31]. Another study in type 2 diabetic patients showed that increased levels of urinary albumin excretion, but not reduced estimated glomerular filtration rate, were associated with increased arterial stiffness, evaluated by carotid–femoral PWV, after multivariate adjustments [32].

Recently, we have reported on the determinants of increasing or reducing (“de-stiffening”) aortic stiffness on a long-term follow-up study with two carotid–femoral PWV measurements performed over a median of 4.2 years [45]. The main determinants of reducing or persisting with low aortic stiffness during follow-up were a better glycemic and blood pressure control and a lower heart rate. However, the presence of microvascular complications at baseline, particularly retinopathy and nephropathy (microalbuminuria), were independently associated with aortic stiffness worsening, together with older age and female gender [45]. This suggests that the presence of microvascular complications might prevent aortic stiffness improvement despite optimal metabolic and blood pressure control in high-risk patients with type 2 diabetes.

On the other hand, longitudinal studies investigating the relationships between aortic stiffness and the future occurrence of microvascular diabetic complications are scarce [33,34]. Relevantly, two longitudinal studies, both using carotid–femoral PWV as the method of evaluation of central aortic stiffness, demonstrated the prognostic importance of increased aortic stiffness at baseline for the prediction of future development and progression of diabetic nephropathy [33] and of peripheral neuropathy [34] in type 2 diabetic patients. The first study included 461 Japanese patients followed up for 6 years, and demonstrated a significant independent association between baseline carotid–femoral PWV and incident abnormal albuminuria and also with the rate of annual decline in glomerular filtration rate [33]. The second one included 477 high-risk type 2 diabetic patients followed up for 6 years, and showed that increased aortic stiffness predicted future development and progression of diabetic peripheral neuropathy, independent of metabolic control [34]. Patients with increased aortic stiffness, defined by a carotid–femoral PWV > 10 m/s, had a two-fold higher incidence of installation or worsening of diabetic peripheral neuropathy than those with lower aortic stiffness (incidence rate ratio: 1.96; 95% confidence interval: 1.18–3.23), after adjustments for several diabetes-related covariates, including metabolic and blood pressure control. These studies in conjunction suggest that there may be potential physiopathological connections between macro- and microvascular alterations in type 2 diabetes.

## 4. Possible Physiopathological Links between Aortic Stiffness and Micro- and Macrovascular Diabetic Complications

The physiopathological mechanisms associating augmented aortic stiffness to cardiovascular outcomes remain largely unsettled. The most accepted hypothesis is that a high aortic stiffness causes a precocious backward pulse wave return during systole and consequently higher systolic and lower diastolic central pressures. This phenomenon provokes an augment in left ventricular workload, and subsequent left ventricular hypertrophy and a reduction in coronary perfusion, potentially causing myocardial ischemia. However, this assumption has been disputed because of the fact that aortic PWV has been demonstrated to be an important prognostic cardiovascular risk marker, but not a measure of central aortic pressure or of backward pulse wave reflection [39]. Alternatively, increased aortic stiffness may represent the accumulated damage to the arterial wall due to aging, genetic influence and to other cardiovascular risk factors over time [2]. If so, it can be a joining measurement of the total cardiovascular risk factor burden over time on the vascular system, and hence a better cardiovascular risk estimate than isolated single measurements of each cardiovascular risk factor. Indeed, increased aortic stiffness is currently considered as an intermediate preclinical stage of cardiovascular disease development [46]. Moreover, augmented arterial stiffness has been considered an important marker of vascular ageing, and ageing has been consistently associated with endothelial dysfunction, insulin resistance, type 2 diabetes development and future occurrence of clinical cardiovascular diseases [47,48,49,50,51]. Hence, insulin resistance at vascular level, by inducing endothelial dysfunction, may increase vascular ageing and arterial stiffness, leading to higher risk of cardiovascular events occurrence [49,50,51].

On the other hand, the potential physiopathological mechanisms linking increased aortic stiffness to diabetic microvascular disease development is still mainly speculative. As discussed before, the presence of diabetic microvascular complications is a predictor of future worsening of aortic stiffness [45], whereas pre-existent increased aortic stiffness predicts the future development of diabetic peripheral neuropathy [34] and nephropathy [33]. This suggests that the underlying physiopathological mechanisms may be bidirectional. Impaired vasodilatation of small arteries due to endothelial dysfunction associated with diabetic microvascular disease may increase backward pulse wave reflection and central aortic pulse pressure, with subsequent damage to the central arteries wall. In other words, microvascular disease may determine the injury of large arteries by an inward remodeling mechanism [1]. Otherwise, increased aortic stiffness can itself harm the microvessels by leading to an increased transmission of larger injurious pulsatile pressure waves to microcirculation due to a loss of the normal aortic buffering property. [1,2,4]. In healthy young individuals, the muscular peripheral arteries are much stiffer than central arteries, and this stiffening gradient protects the microcirculation [1,17]. Patients with diabetes had a preferential stiffening of central arteries, with less effect on muscular arteries [15,20]. This leads to loss of the normal stiffness gradient between central and peripheral arteries, called impedance matching, which leads to increased transmission of this enhanced, potentially hazardous, forward pulsatile pressure wave to the microcirculation. This mechanism may be particularly harmful in organs with higher blood flows and lower vascular resistances, such as the central nervous system (retina) and kidney [1,2,4,17], favoring the development of diabetic retinopathy and nephropathy.

Another potential explanation linking increased arterial stiffness to diabetic microvascular complications development is that they may share common physiopathological pathways. Two physiopathological pathways emerge as potentially involved in this association: advanced glycation end-products (AGEs) formation and renin–angiotensin system overactivation. Increased aortic stiffness is accepted to be related to quantitative and qualitative changes in the arterial wall content of elastin and collagen [1,2]. Evidence implies that such modifications may be due not solely to short-term hyperglycemia, but also to carbonyl and oxidative stress, chronic inflammation and endothelial dysfunction, encompassing that induced by chronic hyperglycemia and formation of AGEs [17]. Increased aortic stiffness has been associated with hemoglobin A1c (HbA_1c_) levels in cross-sectional investigations [52,53], and as discussed previously, we have demonstrated that a better glycemic control was associated with the attenuation of aortic stiffness in type 2 diabetic patients [45]. This suggests that there could be causal relationships between HbA_1c_ levels and aortic stiffness progression/regression, which may be in part intermediated by AGEs formation. Long-term hyperglycemia augments the reaction between glucose and proteins and yields cross-linking of collagen, elastin and other molecules, so-called AGEs, which have been demonstrated to promote collagen accumulation, tissue inflammation and fibrosis within the wall of the blood vessels [52]. Long-term hyperglycemia may, in addition, influence the arterial wall by launching proliferation of smooth muscle cells [54]. Furthermore, an investigation demonstrated a decrease of arterial stiffness using composites that influence or rupture the structure of AGEs, which may constitute a future treatment option [55]. Giving further evidence that long-term hyperglycemia and higher HbA_1c_ values may be associated with an increase in aortic stiffness, pentosidine levels (a well-delineated AGE) were independently related to the progression of aortic stiffness in diabetic patients on hemodialysis followed up for 1.2 years [56].

Otherwise, the renin–angiotensin system (RAS) is over-activated at the vascular wall in conditions of aging, atherosclerotic alterations and in diabetes [57]. RAS activation may increase arterial stiffness through its powerful vasoconstrictor, angiotensin II, which has hypertrophic and conceivably hyperplastic effects on vascular smooth muscle cells and cardiomyocytes and augments extracellular matrix production [58]. Data from clinical and experimental investigations have implied that the activation of the RAS may contribute to the progression of arterial stiffness. Anti-hypertensive medications that promote RAS blockade reduce more arterial stiffness than other anti-hypertensive drug classes, in patients with hypertension or with other cardiovascular conditions [58,59,60,61]. Local RAS activation also has physiopathological implications on diabetic microvascular disease development and progression, particularly in nephropathy [62] and in retinopathy development [63]. Moreover, the strong renal protective effect of RAS blockers in diabetic nephropathy has been extensively demonstrated [64].

**Figure 1:**
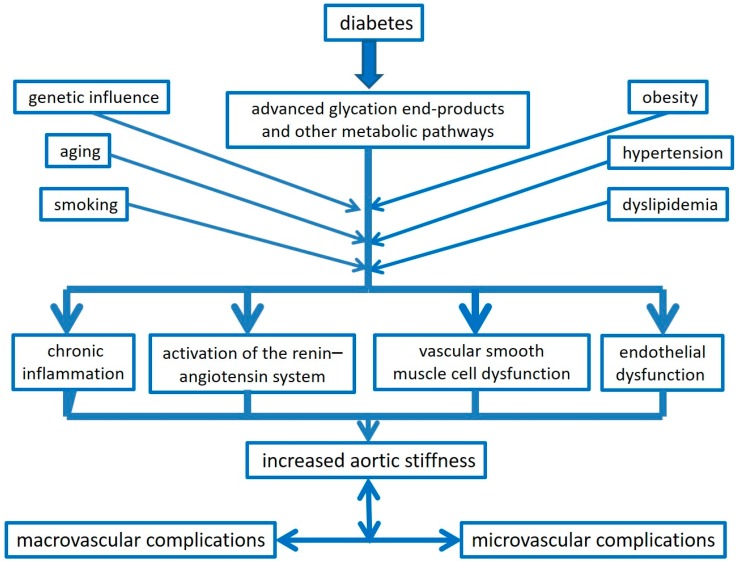
Schematic representation of the potential physiopathological pathways involved in the relationships between increased aortic stiffness and micro- and macrovascular complications in diabetes.

Figure 1 summarizes the potential physiopathological mechanisms underlying the relationships between increased aortic stiffness and diabetic micro- and macrovascular complications.

## 5. Possible Interventions Directed at Reducing Aortic Stiffness in Diabetes

There are several potential pharmacological and non-pharmacological interventions aiming to reduce (“de-stiffening”) aortic stiffness. Regarding possible effects of weight loss on arterial stiffness, a meta-analysis involving 1259 patients suggested that modest weight loss (mean 8%) achieved with diet and lifestyle measures may reduce arterial stiffness [65]. Although some studies assessing the effects of exercise have suggested a possible beneficial effect on arterial stiffness in type 2 diabetic patients [66,67], others did not [68]. These studies used different techniques and included a small number of patients, making it difficult to draw conclusions on the overall efficacy of exercise on arterial stiffness attenuation. In relation to this subject, a recent meta-analysis showed that currently there is insufficient evidence regarding the efficacy of regular exercise for improving vascular function and arterial stiffness as measured by flow mediated vasodilatation and PWV measurement in type 2 diabetes [69]. Based on the fact that arterial stiffness, measured as carotid–femoral PWV, is a strong prognostic factor, independent of classic risk factors, and that it could be an important treatment target, several studies investigated whether different nutritional and pharmacological interventions could reduce arterial stiffness independently of their effect on blood pressure or metabolic effects [70,71,72]. However, it is still unclear whether reducing arterial stiffness may improve the prognosis beyond treatment of standard cardiovascular risk factors. To date, only one study in end-stage renal failure patients demonstrated that aortic stiffness attenuation was associated with improved survival [73].

## 6. Conclusions and Final Remarks

Aortic stiffness is increased in patients with type 2 diabetes and it is related to the presence of diabetic micro- and macrovascular complications. Further, it has been demonstrated to be an independent predictor of worse cardiovascular outcomes in type 2 diabetic patients and also a risk marker for diabetic nephropathy and peripheral neuropathy onset and progression. However, the physiopathological mechanisms linking increased aortic stiffness and the occurrence of diabetic vascular complications still remain unclear. Based on current evidence, we recommend routinely evaluating aortic stiffness by its gold standard method, the carotid–femoral PWV measurement, which is a relatively simple, moderate-cost, and well-standardized procedure. Evidence that optimal glycemic and blood pressure control is associated with attenuation/prevention of progression of aortic stiffness needs to be confirmed on future randomized studies, as well as whether other interventions can reduce aortic stiffness and improve morbidity and mortality in patients with type 2 diabetes.

## Figures and Tables

**Table 1:** Main studies evaluating associations between aortic stiffness and diabetic microvascular complications.

Reference	Number of Patients	Study Design	Microvascular Complication Evaluated	Arterial Stiffness Index	Main Findings
[19]	600	Cross-sectional	Retinopathy	AIx	Augmentation index was independently associated with the presence of retinopathy.
[26]	494	Cross-sectional	Retinopathy	hf-PWV	Aortic stiffness was independently associated with retinopathy.
[23]	294	Cross-sectional	Peripheral neuropathy	ba-PWV	Increased arterial stiffness was independently associated with peripheral neuropathy.
[21]	731	Cross-sectional	Peripheral neuropathy	CAVI	Increased arterial stiffness was independently associated with peripheral neuropathy.
[22]	692	Cross-sectional	Peripheral neuropathy	ba-PWV	Increased arterial stiffness was independently associated with peripheral neuropathy.
[24]	45	Cross-sectional	Cardiovascular autonomic neuropathy	cf-PWV	Aortic stiffness correlated with autonomic neuropathy.
[29]	306	Cross-sectional	Microalbuminuria	ba-PWV	Arterial stiffness was independently associated with microalbuminuria.
[18]	167	Cross-sectional	Microalbuminuria	cf-PWV	Aortic stiffness was independently associated with increased albuminuria.
[27]	134	Cross-sectional	Nephropathy	cf-PWV	Aortic stiffness was associated with albuminuria without adjustment for blood pressure, and with lower eGFR.
[28]	614	Cross-sectional	High–normal albuminuria	cf-PWV	Aortic stiffness was independently associated with higher albuminuria even in the normal range.
[31]	122	Cross-sectional	End-stage renal disease	cf-PWV	Aortic stiffness was independently associated with reduced renal function.
[32]	706	Cross-sectional	Microalbuminuria	cf-PWV	Increased aortic stiffness was independently associated with microalbuminuria, but not with reduced eGFR.
			Reduced renal function		
[20]	482	Cross-sectional	Retinopathy	cf-PWV	Increased aortic stiffness was independently associated with nephropathy, retinopathy and peripheral neuropathy.
			Nephropathy		
			Peripheral neuropathy		
			Cardiovascular autonomic neuropathy		
[30]	320	Cross-sectional	Albuminuria	CAVI	Increased arterial stiffness was independently associated with albuminuria and peripheral neuropathy, but not with retinopathy.
			Retinopathy		
			Peripheral neuropathy		
[25] *	635	Cross-sectional	Albuminuria	cf-PWV	Increased aortic stiffness was independently associated with increased albuminuria, retinopathy and cardiac dysautonomia.
			Retinopathy		
			Cardiovascular autonomic neuropathy		
[33]	461	Prospective	Microalbuminuria	cf-PWV	Aortic stiffness independently predicted incident microalbuminuria and was associated with the rate of decline in eGFR.
			Reduced renal function		
[34]	477	Prospective	Peripheral neuropathy	cf-PWV	Increased aortic stiffness independently predicted the future development and progression of peripheral neuropathy.

* The only study with type 1 diabetic patients. All others were on type 2 diabetes. Abbreviations: AIx, augmentation index; hf-PWV, heart–femoral pulse wave velocity; ba-PWV, brachial–ankle pulse wave velocity; CAVI, cardio-ankle vascular index; cf-PWV, carotid–femoral pulse wave velocity; eGFR, estimated glomerular filtration rate.
